# Preliminary Evidence of Chlamydiosis in Koalas of the Greater Geelong Region, Victoria: A Potential Emerging Threat?

**DOI:** 10.3390/ani15142048

**Published:** 2025-07-11

**Authors:** Gianna Kramer, Janine Duffy, Valentina S. A. Mella

**Affiliations:** 1 Sydney School of Veterinary Science, The University of Sydney, Sydney, NSW 2006, Australia; 2 Koala Clancy Foundation, P.O. Box 20, Bacchus Marsh, VIC 3340, Australia

**Keywords:** *Phascolarctos cinereus*, chlamydiosis, disease presence, conservation, wildlife management

## Abstract

Koalas (*Phascolarctos cinereus*) face many threats, with one of them being the disease chlamydiosis caused by the bacterium *Chlamydia pecorum*, a cause for infertility and one of the main causes of population decline. In New South Wales, Queensland, and the Australian Capital Territory, the koala is an endangered species, but in Victoria, koalas are widespread and overabundant in some areas. The koala populations in the You Yangs Regional Park (YYRP) and Brisbane Ranges National Park (BRNP) in Victoria are reported to be in decline, although there have been no studies looking into population density or disease. The aim of this study was to investigate the presence of *C. pecorum* in the koala populations of the YYRP and BRNP to understand whether disease may be a cause of concern. DNA was extracted from scats, and qPCR was used to detect the presence and load of *C. pecorum*. *C. pecorum* was found in 12/18 (66.7%) scat samples. Clinical signs of disease, age, and sex did not affect infection shedding load. These results confirm the presence of chlamydiosis within the YYRP and BRNP koala populations. More detailed studies are recommended to quantify prevalence of the disease in these populations and to understand the consequences of chlamydiosis in these areas.

## Simple Summary

Chlamydial disease is a major contributor to the decline in koala populations in Australia. In the states where koalas are listed as being endangered, chlamydiosis is closely monitored in many populations. In Victoria, however, where the species is considered overabundant, management practices differ, and less research has been conducted to monitor the presence of chlamydiosis. Populations of koalas in the You Yangs Regional Park and Brisbane Ranges National Park in Victoria have been declining, but the reasons behind this remain unexplored. The objective of our study was to assess the presence of disease within these two koala populations. We found that 66.7% of koalas tested positive for *Chlamydia pecorum*, providing a possible explanation for the observed population decline. This finding highlights the importance of disease surveillance in Victorian koala populations to prevent potential dramatic declines similar to those observed in other states and to inform the development of effective management strategies.

## 1. Introduction

Koalas (*Phascolarctos cinereus*) have a complex conservation status across Australia [1]. The species is declining in New South Wales (NSW), Queensland, and in the Australian Capital Territory, primarily due to deforestation and disease, and is listed as endangered in these jurisdictions under federal legislation [2]. In contrast, koalas in Victoria are considered secure [3] and occur at high densities (≥2 koalas per hectare) in some regions, often to the point of overabundance, particularly in island populations or areas with poor habitat connectivity and limited dispersal opportunities [4,5]. These localised high-density populations have historically contributed to the perception that koalas are abundant throughout the state. However, their distribution across Victoria is uneven and shaped by extensive historical clearing and habitat fragmentation, and robust population data are lacking for much of their range [6,7]. Management strategies for the species in Victoria in areas with high-density populations include translocation and fertility control [3,8,9] to mitigate the impacts of localised over-browsing [10], nutritional stress, and in extreme cases, koala mortality [5,11].

The most significant disease for koalas is chlamydiosis, caused by the Gram-negative bacterium *Chlamydia pecorum* which replicates within cells [12]. Infection can be established in many different body systems, including the eye, where it can cause conjunctivitis, leading to scarring and blindness; the urinary tract, causing cystitis; and the reproductive tract, causing salpingitis and endometritis in females and epididymitis and orchitis in males [13,14], which can result in scarring and infertility in both sexes [13]. Chlamydiosis impacts koalas differently across regions, with Victoria seemingly experiencing lower infection rates [15], while other states face more severe outbreaks, contributing to population declines and increased conservation concerns [16]. Some populations of koalas in NSW and Queensland have up to a 94% infection rate, causing irreversible infertility [17,18] and high mortality in free-ranging individuals [19]. Compared to these populations, the incidence and severity of ocular and urogenital clinical signs are often decreased or absent in Victorian populations [20]. Nevertheless, chlamydiosis has contributed to local population declines in Victoria, including Walkerville, Phillip Island, and the Ballarat region [9,10,21]. The combined prevalence of *C. pecorum* in populations from South Gippsland, Raymond Island, Cape Otway, and Mallacoota was 49% in 2018 [22].

The koalas of the You Yangs Regional Park (YYRP) and Brisbane Ranges National Park (BRNP) in Victoria are regularly monitored by citizen scientists of the Koala Clancy Foundation (https://www.koalaclancyfoundation.org.au/). A decline in numbers has recently been observed in the populations (unpublished data), reflecting general declines in the region [23], and chlamydial clinical signs have been observed, but disease presence has not been confirmed. Previous studies of koalas in these areas focused on individual recognition by nose markings [24], natural drinking behaviour [25], and investigations into how koalas stay hydrated [26]. However, there is no information on the presence of disease in these locations. Monitoring disease in wildlife is vital to be able to manage its spread and impact [27]. This is especially important in Victoria, where management strategies are developed with secure populations in mind, but the true status of some is unknown due to limited monitoring and field research [28]. This may result in population declines going unnoticed and the increasing conservation significance of koalas in Victoria. Understanding the regional variations in chlamydiosis in koalas is crucial for developing effective conservation strategies given that the disease’s impact differs significantly across populations in different areas.

The use of scats to detect *C. pecorum* within a koala population is non-invasive and cost effective and can be carried out on a large scale [29]. The tests have 100% specificity, while sensitivity varies depending on the clinical presentation of disease, with scats from individuals with clear clinical signs of urogenital disease having 78% sensitivity, and those from koalas without obvious clinical signs having a sensitivity of 58% [29]. Despite its low sensitivity, this method is practical and allows for an initial investigation of disease in areas where chlamydiosis has never been investigated. The aim of this study was to evaluate the presence of *C. pecorum* in koalas from the YYRP and the BRNP through the isolation of chlamydial DNA from scats. Monitoring and maintaining healthy populations may prevent Victorian koalas from facing the same 2050 extinction predictions of NSW koalas [30].

## 2. Materials and Methods

### 2.1. Sample Collection

**Figure 1:**
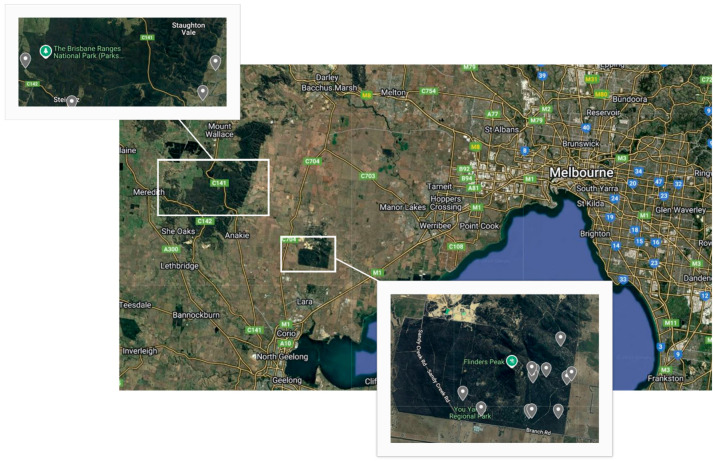
Study sites and locations of koala (*Phascolarctos cinereus*) scat collections in the You Yangs Regional Park (**bottom**) and Brisbane Ranges National Park (**top**), Victoria, Australia.

Koala scats were collected opportunistically between October and November 2023 from the YYRP and the BRNP in the Greater Geelong Region of Victoria (Figure 1) during regular individual koala monitoring by the Koala Clancy Foundation (https://www.koalaclancyfoundation.org.au/). At the time of collection, the koalas were identified from the ground using the nose pattern identification technique [24], and any information from previous regular monitoring of the individuals was recorded, including sex, estimated age, and breeding history if female. Presence or absence of clinical signs of disease, such as conjunctivitis or wet bottom, were also recorded if visible. Scats were collected using scat collection kits (Integrated Sciences) only if fresh (i.e., covered in mucus and shiny) and if deposited while koalas were observed. Samples were then kept frozen at −18 °C until shipped overnight to the Koala Health Hub (KHH) at the Sydney School of Veterinary Science at the University of Sydney to be analysed.

### 2.2. DNA Extraction and Quantitative Polymerase Chain Reaction (qPCR) Analysis

DNA was extracted from scat samples using the ISOLATE Faecal DNA Kit (Bioline, Memphis, TN, USA). Thawed samples were vortexed, and a mixture of collection solution and scat pellet was homogenised using FastPrep™ (MP Biomedicals, Santa Ana, CA, USA) and then centrifuged. The supernatant underwent further centrifugation and was filtered through a Spin II A Filter (MP Biomedicals, Santa Ana, CA, USA). After adding Faecal DNA Binding Buffer, the mixture was processed through a spin column with sequential wash steps. DNA was then eluted, passed through a Spin II B Filter pre-treated (MP Biomedicals, Santa Ana, CA, USA) with Faecal Prep Solution, and centrifuged to obtain the final extracted DNA. The extracted DNA was then processed using the CFX Opus 96 Real-Time PCR System (Bio-Rad, Hercules, CA, USA). Quality controls were included alongside the test samples and underwent the same extraction and analysis procedures. These included blank extractions made during each batch extraction of DNA to ensure no cross contamination occurred during handling. A PCR positive control and a PCR negative control were included to ensure the PCR reaction was working and to check for the absence of contamination. A multiplex, TaqMan^®^ (Thermo Fisher Scientific, Waltham, MA, USA) probe assay was used to test for the presence of koala DNA via beta-actin mRNA, *C. pecorum* (ompB), and *Chlamydia* genus (23S rRNA). These specific DNA sequences were used as they are highly conserved targets over all genotypes.

qPCR results were interpreted by first looking at the beta actin levels to ensure adequate levels of koala DNA were present in the samples (Table 1). Samples lacking adequate beta-actin levels were considered as failing quality control and excluded from analysis. This was followed by the interpretation of *Chlamydia* genus and *C. pecorum* levels to differentiate samples into four categories, namely negative (no *C. pecorum* detected), positive (*C. pecorum* DNA was clearly detected), weak positive (*C. pecorum* DNA was detected but at a low level), and suspect (*C. pecorum* could not be definitively excluded), depending on their cycle threshold (CT) values (Table 1). For the purposes of analysis, we took a conservative approach and considered all weak positives and suspect results as positive. To quantify the amount of chlamydial shedding, ΔCT values were then calculated by subtracting the CT of *C. pecorum* from the CT of the beta-actin housekeeping gene (to account for quality of swab yield), with higher chlamydial shedding being represented by a lower ΔCT value (as in [31]).

### 2.3. Statistical Analysis

All analyses of data were performed using R, version 4.2.2 [32]. A Q-Q plot was generated using the ‘qqnorm’ function to ensure data were normally distributed. We then used a linear model using the package ‘lme4’ [33] to test the effects of sex, age, and urogenital and ocular signs on *C. pecorum* infection shedding severity (using ΔCT values). Location could not be included in the models as all samples collected from Brisbane Ranges National Park were positive for *C. pecorum*.

## 3. Results

A total of 19 samples (15 from the YYRP and 4 from the BRNP) were collected from individual koalas (8 females, 10 males, and 1 of unknown sex). All koalas were adults (age range of 3–12 years) except for one dependent joey of around 10 months of age. Three koalas had clinical signs of *C. pecorum* infection in both the ocular area (i.e., conjunctivitis) and urogenital tract (i.e., perineum staining) upon observation at the time of scat collection. Two koalas had ocular signs only, and the rest of the koalas had no clinical signs or were unable to be evaluated from the ground. All samples except 1 had successful amplification: 6/18 (33.3%) samples tested negative for *C. pecorum*, 6/18 (33.3%) tested positive, 5/18 (27.8%) were weak positives, and 1/18 (5.6%) was suspect positive.

There was no effect of sex (F_1,3_ = 0.014, *p* = 0.914) and age (F_1,3_ = 0.039, *p* = 0.857) on *C. pecorum* shedding severity. The presence of ocular (F_1,3_ = 0.149, *p* = 0.725) and urogenital (F_1,3_ = 0.676, *p* = 0.471) signs of chlamydiosis also had no effect on *C. pecorum* shedding status.

## 4. Discussion

This study investigated the presence of *C. pecorum* infection in a limited number of koalas from the BRNP and the YYRP, Victoria, where testing had not been conducted previously, using non-invasive DNA scat analysis. Five koalas presented clinical signs of *C. pecorum* upon observation at the time of scat collection, including five with ocular signs and three showing urogenital signs. We found that 66.7% of the samples tested positive for *C. pecorum*, with all individuals from the BRNP and 8/15 of koalas from the YYRP being infected. The presence of *C. pecorum* within these populations may have been historical, as koalas were translocated to the BRNP in the 1940s from Philip Island, Victoria, where *C. pecorum* infection was already present [4]. However, koalas located near Warrnambool, in the South West Coast region, Victoria, from the same island source population, had only a 7% prevalence of *C. pecorum* and no observable loss of fertility [8], indicating that the disease may be recent in some areas. Hence, it is possible that the animals became infected at the location where they were translocated [9]. The high rate of disease we found was unexpected, as previous studies between 2015 and 2018 found that infection in Victorian koala populations was lower (1–49%) than the rate found in our study [15,20,22]. This increased disease rate may have been facilitated by different factors, including stress [34] and koala retrovirus (KoRV), with higher viral loads being positively correlated with the severity of chlamydiosis in southern koalas [35,36]. Habitat quality is decreased in the YYRP with invasive weeds such as boneseed (*Chrysanthemoides monilifera*) and bridal creeper (*Myriophyllum asparagoides*) covering extensive areas of the park and impacting the local flora and fauna by preventing the growth of native plants [37,38], including eucalypt species, potentially reducing the regrowth of trees used by koalas. The YYRP and BRNP are also surrounded by extensive agricultural land and are neighbouring several sand mines, limiting the movement of koalas and potentially resulting in increased stressors [16,34]. As the YYRP is situated within a rain shadow of the Otway Ranges (www.parks.vic.gov.au, accessed on 16 April 2025), climatic conditions, including droughts and heatwaves experienced in the area, may also compound the prevalence of disease in koalas. A recent study found that weather can affect koala immune function and increase the expression of stress receptor pathways, possibly increasing susceptibility to infection [39].

The sensitivity of the DNA detection of *C. pecorum* in scats can be dependent on the presence of clinical signs, with tests from individuals less affected by clinical disease, showing a lower sensitivity [29]. We did not observe severe clinical signs in any of the koalas in this study. However, Victorian koalas carry *C. pecorum* ompA genotype B, which differs from the genotype found in northern koalas [15,40], where the use of scats for *C. pecorum* detection was developed [29]. This appears to result in milder clinical signs [15,20], which would increase the likelihood of false negatives when using scats to test for *C. pecorum* within a population [41], potentially leading to an underestimation of the true disease burden in our study. This does not lower the value of using scats to test for *C. pecorum* as a preliminary exploratory tool for the disease as this method does not require invasive capture and handling, which would be required to gather direct ocular and urogenital swabs.

Females and males appeared equally susceptible to chlamydiosis in our study areas as we found no effect of sex on the shedding rate of *C. pecorum*. Our sample size was limited, but other studies conducted in NSW [31], Queensland [42], South Australia [35], and Victoria [20] also showed no effect of sex on shedding and/or the infection rate of individuals. This result can probably be ascribed to the sexually transmitted nature of *C. pecorum* disease, with sexually mature males and females both spreading the infection during mating season [16]. However, other studies found that females were more likely to show a higher incidence of visible signs of urogenital infection than males [31,43]. More samples from individuals with observable clinical signs may be needed from the YYRP and BRNP to be able to detect a relationship between sex and urogenital tract disease within these populations.

Estimated age also showed no effect on *C. pecorum* shedding, consistent with previous studies [20,42]. In koalas, mature age can sometimes be associated with an increased likelihood of *C. pecorum* infection due to the sexually transmitted nature of this disease [16]. However, in this study, we sampled only adult koalas that were sexually mature (except a joey which was sampled opportunistically while its mother was sampled), and these koalas did not differ much in shedding load and clinical signs, showing that the disease can affect sexually active individuals of different ages in similar ways [15].

All of the females with high historical fecundity tested positive for chlamydiosis, and the female who had the highest shedding load (i.e., the lowest ΔCT value) was also the one with the highest number of joeys recorded in the past, indicating that chlamydial infections in the YYRP may be recent. It is also possible that recent infections involve a different and more virulent *Chlamydia* genotype [44], particularly if they are associated with decreased fecundity. This may have contributed to the initial observations of population declines. Chlamydiosis is generally linked to reduced reproductive rates in females [35], and the decrease in fertility observed in female koalas is often due to reproductive tract changes, including unilateral or bilateral ovarian cysts, changes in uterus appearance, and associated structures [16]. These changes are often not apparent or identifiable without the use of ultrasound or abdominal palpation [16]. Even in areas where reproductive rates are very low, reproductive disease signs in females are sometimes undetectable [42]. Further investigations of reproductive tract pathology in female koalas of these populations would provide valuable insights into the structural impacts of *C. pecorum* infection in these areas.

## 5. Conclusions

This study has identified chlamydial disease for the first time in these populations and found a higher-than-expected infection rate, potentially responsible for the decline in koala numbers observed in these areas. In this initial investigation, we had a small sample size (*n* = 18), which prevented us from investigating disease variations between the BRNP and YYRP populations. Future studies should aim to include a greater number of samples to improve the robustness of the findings. Our study confirms that the scat analysis method is a valuable primary assessment tool to detect *C. pecorum* in populations where chlamydial disease status is unknown. The next step is to carry out a large-scale population disease assessment, including the capture and close examination of koalas.

Our findings are consistent with the observed decline in koala numbers around Australia and the increased prevalence of *C. pecorum* infection within many koala populations. Further investigations into the disease in these important koala populations would be beneficial. Longitudinal studies would allow for the progression and impact of *C. pecorum* within these populations to be recorded and for tailored management strategies to be developed.

## Figures and Tables

**Table 1:** The result categories determined by the cycle threshold (CT) value limits of the Quantitative Polymerase Chain Reaction (qPCR) for *Chlamydia pecorum*. Beta-actin is used as a housekeeping gene to account for the quality of the swab yield. Quality control fail (QC-fail) means that a sample did not meet the required quality standards to be included in the analysis.

Beta-Actin	*Chlamydia pecorum*	*Chlamydia* Genus (23S)	Result
CT ≤ 32	no amplification	no amplification	Negative
CT ≤ 32	CT ≤ 34	CT ≤ 34	Positive
CT ≤ 32	CT 34–40	CT 34–40	Weak positive
CT > 32	CT 34–40	CT 34–40	Weak positive
CT ≤ 32	no amplification	CT 34+	Suspect
CT > 32	no amplification	no amplification	QC-fail
no amplification	no amplification	no amplification	QC-fail

## Data Availability

The data that support the findings of this study are available from the corresponding author upon request. There are no restrictions on data availability.

## Abbreviations

The following abbreviations are used in this manuscript:

YYRP	You Yangs Regional Park
BRNP	Brisbane Ranges National Park
DNA	Deoxyribonucleic acid
qPCR	Quantitative polymerase chain reaction
NSW	New South Wales
KHH	Koala Health Hub
PCR	Polymerase chain reaction
mRNA	Messenger ribonucleic acid
ompB	Outer membrane protein B
CT	Cycle threshold
ΔCT	Delta cycle threshold
KoRV	Koala retro virus
QC-fail	Quality control fail
